# The Origin of Teratogenic Retinoids in Cyanobacteria

**DOI:** 10.3390/toxins14090636

**Published:** 2022-09-15

**Authors:** Luděk Sehnal, Marie Smutná, Lucie Bláhová, Pavel Babica, Petra Šplíchalová, Klára Hilscherová

**Affiliations:** RECETOX, Faculty of Science, Masaryk University, Kotlarska 2, 61137 Brno, Czech Republic

**Keywords:** retinoids, cyanobacteria, reactive oxygen species, aldehyde dehydrogenases, biosynthesis

## Abstract

Although information about the occurrence and distribution of retinoids in the environment is scarce, cyanobacterial water blooms have been identified as a significant source of these small molecules. Despite the confirmed presence of retinoids in the freshwater blooms dominated by cyanobacteria and their described teratogenic effects, reliable identification of retinoid producers and the mechanism of their biosynthesis is missing. In this study, the cultures of several taxonomically diverse species of axenic cyanobacteria were confirmed as significant producers of retinoid-like compounds. The consequent bioinformatic analysis suggested that the enzymatic background required for the biosynthesis of all-trans retinoic acid from retinal is not present across phylum Cyanobacteria. However, we demonstrated that retinal conversion into other retinoids can be mediated non-enzymatically by free radical oxidation, which leads to the production of retinoids widely detected in cyanobacteria and environmental water blooms, such as all-trans retinoic acid or all-trans 5,6epoxy retinoic acid. Importantly, the production of these metabolites by cyanobacteria in association with the mass development of water blooms can lead to adverse impacts in aquatic ecosystems regarding the described teratogenicity of retinoids. Moreover, our finding that retinal can be non-enzymatically converted into more bioactive retinoids, also in water, and out of the cells, increases the environmental significance of this process.

## 1. Introduction

Retinoids are fat-soluble hormones essential for important developmental processes and homeostasis in vertebrates and invertebrates [1,2]. Retinoid signaling during these processes is mediated through nuclear receptors, the retinoic acid receptor (RAR), and the retinoid X receptor (RXR) [3,4,5]. Compounds interacting with these receptors are called retinoid-like compounds that encompass specific retinoid molecules, as well as other molecules such as β-apocarotenoids [6]. Among various retinoid-like molecules, all-trans retinoic acid (ATRA) has been recognized as the main driver of retinoid signaling. Importantly, excess of ATRA during embryonal development causes abnormalities in many vertebrate species [7]. 

During the last decade, freshwater blooms dominated by cyanobacteria have been identified as the environmental source of retinoids. Pioneering studies [8,9,10] brought the first information about the production of retinal (RAL), ATRA, and its derivatives by freshwater blooms dominated by cyanobacteria *Microcystis*. These studies emphasized the high production of RAL by species from this cyanobacterial genus and determined the retinoid-like activities (potency of sample to interact with retinoic acid receptors) of the blooms’ biomass extracts as well as the surrounding water. Consequently, a recent study [11] revealed that retinoids are produced by freshwater blooms with a diverse taxonomic composition, and without the presence of the *Microcystis* species. The same study expanded our knowledge about the occurrence of other bioactive ATRA derivatives, such as all-*trans* 5,6-epoxy retinoic acid (5,6epoxy-ATRA) or all-trans 4-keto retinoic acid (4keto-ATRA), and their contribution to the total detected retinoid-like activity. Concentrations of these compounds almost completely explained the retinoid-like activity detected in some freshwater bloom samples; however, the same compounds explained less than 1% in the case of other freshwater bloom samples. Importantly, the observed concentrations of retinoids in freshwater bloom biomasses, as well as in the surrounding water, can reach levels causing teratogenic effects in vivo [12,13].

The first Information about the association between retinoid-like compounds and teratogenic effects was reported for laboratory cultures of cyanobacteria [12,14]. Recently, a key role of retinoids in observed teratogenic effects has been reported [13], and importantly, it has also been described for freshwater blooms dominated by cyanobacteria. Although the production of retinoids by cyanobacteria can pose a threat to organisms in the aquatic ecosystems, especially in association with the mass development of cyanobacterial water blooms worldwide, there are huge gaps in our knowledge. Information such as the level of retinoids in other ecosystems and regions across the Earth, the fate of retinoids in aquatic ecosystems, the complete spectra of molecules responsible for observed effects, the key environmental factors affecting their production, or the characterization of retinoids biosynthesis is still missing.

The formation of retinoids is closely related to the biosynthesis of carotenoids. Almost two decades ago, the cyanobacterial enzyme apocarotenoid cleavage oxygenase (ACO) was recognized as an enzyme-catalyzing biosynthesis of RAL from apocarotenoids [15,16]. The consequent bioinformatic study confirmed the widespread distribution of the ACO across cyanobacterial lineage, thus verifying enzyme responsibility for the production of RAL [17]. Although the enzymatic pathway for the production of RAL was shown, the enzymatic basis for most other, biologically active, retinoids, such as ATRA, 4keto-ATRA, 5,6epoxy-ATRA, and 9/13cis-RA, has not been elucidated so far. In vertebrates, aldehyde dehydrogenases were identified as the main enzymes converting RAL to ATRA. Nevertheless, it is the only group of organisms where retinoic acid biosynthesis is described in detail. In cyanobacteria, one study [18] reported a possible RAL-RA converting enzyme; however, the study lacks important experimental validation of the results, such as enzyme-substrate specificity tests, product concentration, or standard enzymatic kinetics.

Next to enzymatic processes, non-enzymatic processes also play an important role in the metabolism of carotenoids. Recently, free radical-mediated oxidation of carotenes and xanthophylls has been reviewed [19], and the oxidative action of reactive oxygen species (ROS) has been reported as a significant player in carotenoid metabolism. Biosynthesis of RAL from higher carotenoids provides a nice example of the interconnection of enzymatic and non-enzymatic processes in the metabolisms of carotenoids. Recent research showed that enzymatically synthesized carotenoids can be oxidized by ROS into a series of epoxides and apocarotenoids. Consequently, apocarotenoids containing a C15-C15’ double bond can be enzymatically converted into RAL by cyanobacterial ACOs. Nevertheless, the consequent susceptibility of RAL to oxidation by ROS has not been studied in detail yet.

Here, using methods of bioinformatics, phylogenetics, analytical chemistry, and bioassays, we aimed to illustrate the presence/absence of a genetic basis for enzymatic conversion of RAL to ATRA and its metabolites across cyanobacteria phylum, and to describe the production of the different retinoids from RAL. We analyzed the distribution and phylogenetic relationship of cyanobacterial aldehyde dehydrogenases belonging to the same protein superfamily (SF) as vertebrates’ retinal dehydrogenase. We further investigate the hypothesis that cyanobacterial biosynthesis of ATRA and other RAL metabolites can be mediated via non-enzymatic reactions with ROS. To study RAL oxidation by ROS, we carried out the Fenton reaction, optimized the ratio of individual reagents, and analyzed a spectrum of relevant RAL metabolites together with changes in retinoid-like activity determined in vitro. Based on our results and the current state of knowledge, we illustrated the origin of retinoid production in cyanobacteria encompassing both enzymatic and non-enzymatic processes.

## 2. Results and Discussion

### 2.1. Lab Cultures of Cyanobacteria and Their Retinoids Production

**Figure 1:**
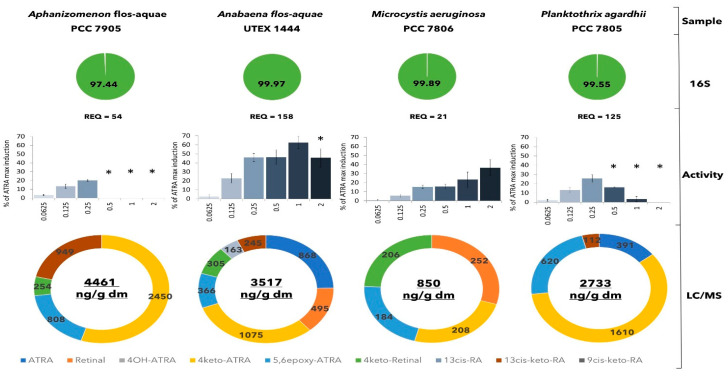
Comparison of retinoid-like activities in axenic laboratory cultures of cyanobacteria. The green circles with a number indicate the percentage representation of specific cyanobacterial species in the sample based on 16S amplicon sequencing. Results of retinoid-like activity are expressed as average ± SD of triplicates. Retinoid-like effects (*y*-axis) are shown as a percentage of maximal induction caused by ATRA. Detected cytotoxicity is indicated by an asterisk (*). Concentrations on *x*-axis are expressed as g dm/L. Values of retinoic acid equivalents (REQs) are expressed as ng ATRA/g dm. The concentration of retinoids in biomass extracts of individual axenic cultures analyzed by LC/MS is expressed in ng/g dm.

Increased attention to the production of retinoids by cyanobacteria has been paid in the last decade. Nevertheless, only one study [10] investigated the production of retinoids in axenic cultures of two coccal cyanobacterial species *Microcystis aeruginosa* PCC 7806 and *Synechococcus sp.* PCC 7002. In addition, the focus of that study was limited to the detection of RAL, ATRA, and 4keto-ATRA. While retinal has low potency to interact with retinoid receptors, ATRA and its derivates elicit a higher potency [20,21]. Importantly, ATRA and its derivates were detected in trace concentrations up to 2.5 ng/g dm and the study did not provide any information about axenicity verification. Thus, although recent studies indicated that cyanobacteria are an important source of retinoid compounds, reliable verification of this fact is still missing, since most studies did not provide any information on the axenicity of the studied cultures. This information is crucial for confirmation of retinoid producers since the presence of enzymes able to convert retinal to ATRA has been also indicated in the bacteria *Bacillus cereus* [22], and there is no information related to other bacteria. Within this study, we examined the retinoid-like activity of extracts of axenic cyanobacterial cultures. Retinoid-like activity and the concentration of individual retinoid compounds, together with axenicity, were characterized for species from taxonomically different genera including *Aphanizomenon, Anabaena, Microcystis,* and *Planktothrix* (Figure 1). Detailed data from the LC-MS/MS analysis of individual retinoids and a complete list of the taxonomic composition of individual cultures based on 16S rRNA gene sequence are available in Supplementary Material S1a and Supplementary Material S1b, respectively.

The highest retinoid-like activity was detected in the biomass extract of heterocystous cyanobacteria *Anabaena* flos-aquae UTEX 1444. The lowest retinoid-like activity was detected in the biomass extracts of coccal cyanobacteria *Microcystis aeruginosa* PCC 7806. Importantly, all axenic cultures were identified as producers of retinoid-like compounds (Figure 1).

These results confirmed that cyanobacteria are the producers of compounds responsible for retinoid-like activity. Moreover, it indicates that cyanobacteria play an important role as a source of retinoid-like compounds also within freshwater blooms, which is especially important in association with the mass development of freshwater blooms worldwide and described teratogenic effects of retinoids on aquatic organisms [20].

### 2.2. Absence of Genetic Basis for ATRA Biosynthesis across Cyanobacterial Genomes

Since vertebrates use the enzyme aldehyde dehydrogenase (aldh) for the biosynthesis of ATRA from RAL, several earlier studies suggested that this mechanism could also be valid for ATRA biosynthesis in cyanobacteria. Specifically, Miles et al. [18] investigated a possible role of specific cyanobacterial cfaldh (CD F1-2) from the species *Chlorogloeopsis fritchii.* Cfaldh is similar to vertebrate retinal dehydrogenase and was proposed as a possible ATRA-producing enzyme. However, our search for this enzyme, aldh (CD F1-2), across cyanobacterial genomes of described retinoid producers did not identify any such enzyme. Moreover, Miles et al. [18] documented that cfaldh is more similar to human ALDH2 (share 17/34 protein signature residues, 68% similarity) than to human ALDH1-A1 (share 9/34 protein signature residues, 64% similarity). It is in concordance with our bioinformatic analysis (Table S1) that showed also a higher similarity of cfaldh to mitochondrial acetaldehyde dehydrogenase from *Homo sapiens*, which belongs to the same superfamily and contains the same CD, but has a different function—the oxidation of acetaldehyde to acetate (Table S2). Thus, a detailed investigation of the function of this enzyme should be performed; for instance, better biochemical characterization of the enzyme or mutant studies can reliably describe the function of this enzyme.

**Figure 2:**
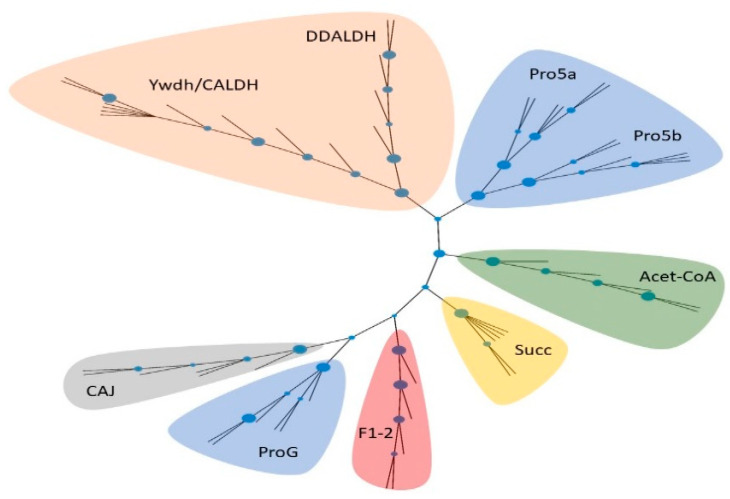
Phylogenetic analysis of cyanobacterial aldh belonging to the same superfamily as retinal dehydrogenase from *Homo sapiens*. Colors indicate different functional groups of studied aldh.

**Figure 3:**
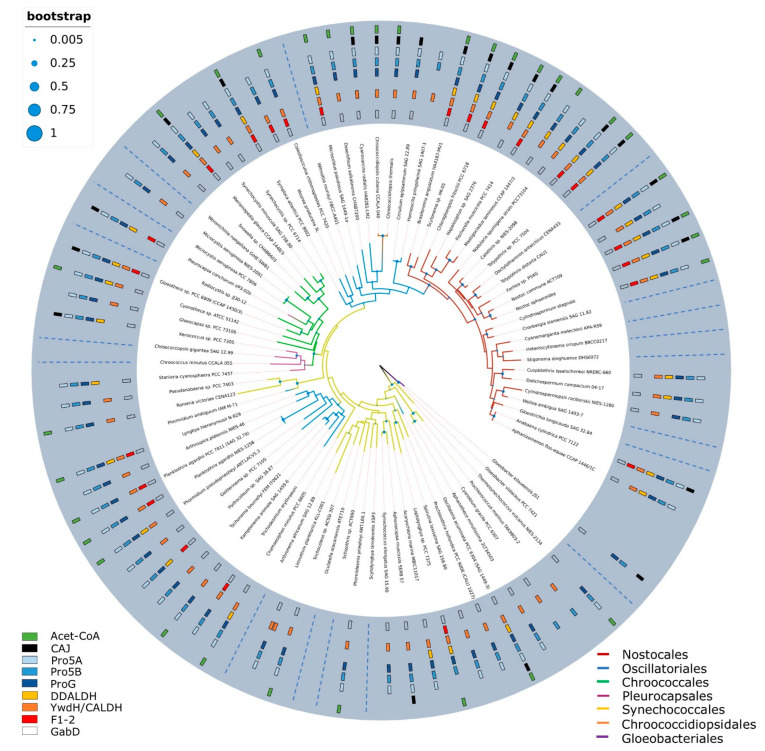
Distribution of cyanobacterial aldh belonging to the ALDH-SF superfamily (containing also retinal dehydrogenase from *Homo sapiens*) across the phylogenetic tree of cyanobacteria. Different cyanobacterial orders are distinguished by different branch colors. The surrounding blue circle provides information about the presence/absence of specific aldh. The distribution of aldh for genera without any available genome is replaced by a dashed line.

To attain a deeper insight into the distribution and evolution of individual aldh across cyanobacterial lineage, we investigated the evolution (Figure 2, Supplementary material S2) and distribution (Figure 3, Supplementary Material S3) of this specific aldehyde dehydrogenase across the available cyanobacterial genomes. First, to clearly define the functional differences between individual types of aldehyde dehydrogenases across cyanobacterial lineage, a phylogenetic analysis was carried out. Our unrooted phylogenetic analysis divided nine groups of enzymes based on a CD into eight separated branches (Figure 2). Importantly, one branch contains enzymes belonging to two differently annotated domains, CALDH and Ywdh. Although the Ywdh domain is curated as a subdomain of CALDH in the NCBI database, we proposed the reannotation of these domains across cyanobacterial genomes into one common domain, since the phylogenetic analysis showed that they constitute orthologous enzymes with a shared evolutionary history across cyanobacterial genomes, and the differences among enzymes are the consequence of speciation.

The analysis of distribution provided information about the conservation of the selected aldh enzymes across cyanobacterial genomes. The results documented the presence of aldh containing CD F1-2 only in 1/3 of the genomes of the studied cyanobacterial genera (Figure 3). Moreover, this enzyme is absent in genomes of all sequenced species from the genera *Microcystis*, *Cylindrospermopsis*, and *Aphanizomenon*, which have been described as producers of ATRA and its derivatives [21]. Therefore, these results along with results of our bioinformatic and chemical analysis strongly indicate that this enzyme is not involved in the metabolism of retinoids in cyanobacteria, at least not universally. Furthermore, the results also provide insight into the distribution of other aldh across the cyanobacterial phylum, where the widespread distribution of dehydrogenases involved in the metabolism of proline (Pro5a, Pro5b), succinate (GabD), and apocarotenoids (CALDH/Ywdh) across cyanobacterial genomes was identified. Since RAL is also an apocarotenoid, apocarotenoid aldh could be considered as a RAL-converting enzyme; however, this aldh involved in apocarotenoid metabolism was shown as incapable of conversion of RAL to ATRA [23].

### 2.3. ROS-Mediated Oxidative Conversion of Retinal to More Bioactive Retinoids

The absence of aldh responsible for the conversion of RAL to ATRA across retinoid producers indicated that ATRA as well as other detected retinoids in cyanobacteria and freshwater blooms can be also produced by another mechanism. A recent review [19] pointed out the significance of free radical-mediated oxidation of carotenoids and emphasized the interplay of enzymatic and non-enzymatic processes in the metabolism of carotenoids. Although radical-mediated oxidation was studied across a range of carotenoids (e.g., [24]), RAL oxidation by ROS with regard to the production of teratogenic retinoids has not been considered so far.

**Figure 4:**
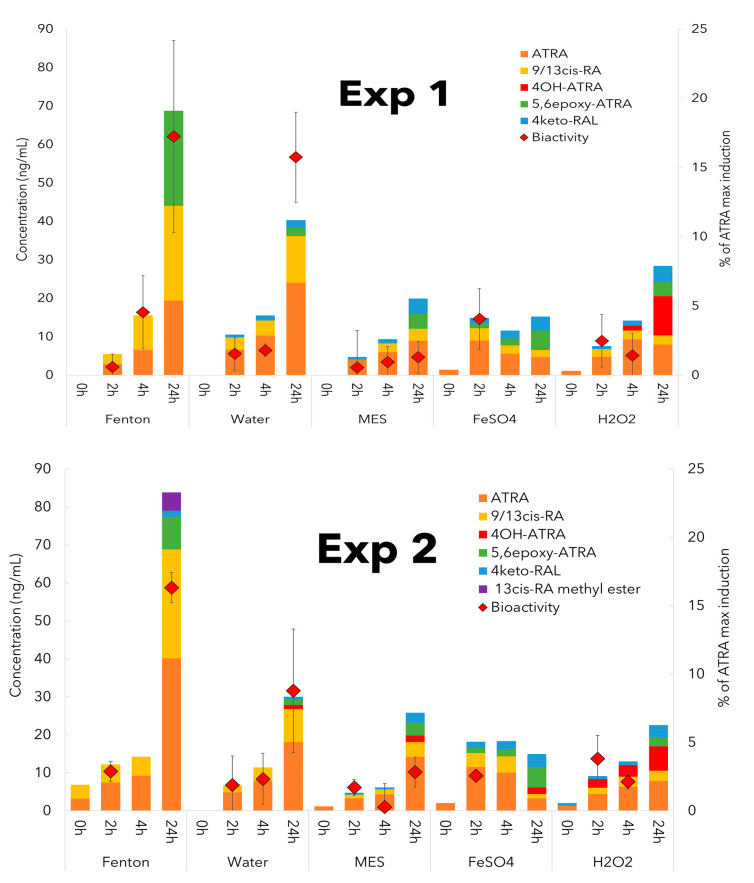
Changes in the concentration of reaction products and bioactivity during oxidation of RAL by reactive oxygen species (ROS) produced in different reaction variants. Concentrations of reaction products are expressed as a cumulative concentration in ng/mL and data are expressed as an average of triplicates. Retinoid-like activities are expressed as % of ATRA max induction and values represent the average from at least three measurements ± standard deviation (SD). The figure shows the results of two independent experiments (Exp1 and Exp2). Abbreviations: Fenton—incubation of RAL with Fenton reaction, Water—incubation of RAL in water, MES—incubation of RAL in MES buffer, FeSO4—incubation of RAL in MES buffer with FeSO4, H_2_O_2_—incubation of RAL in MES buffer with H_2_O_2_.

Our two independent experiments investigating the free radical oxidation of RAL showed a very similar pattern in the levels as well as spectra of the produced compounds (Figure 4). Oxidation of the RAL, a weak agonist of the RAR receptor, resulted in the production of ATRA, 9/13cis RA, 5,6epoxy-ATRA, 4OH-ATRA, 4keto-RAL, and 13cis RA methyl ester. In particular, RAL oxidation products significantly differ in potency to interact with the RAR receptor [20,21], and ATRA elicits approximately 150-fold higher potency to interact with RAR than RAL. Based on the results of oxidation dynamics in time, ATRA was detected as the first and the most abundant product of RAL oxidation observed in our experiments, and other ATRA metabolites seemed to evolve by consequent ATRA oxidation, hydroxylation, or isomerization.

In a detailed look at the spectra of the produced retinoids and their changes in time, different patterns were observed for different tested variants. In all variants except for MES with FeSO_4_, an increase in concentrations of ATRA, 9/13cis RA, and 5,6epoxy-ATRA was recorded during both experiments. These compounds, which represent the molecules with the highest potency to interact with retinoid receptors [21], were detected in freshwater blooms dominated by cyanobacteria [8,10,11] and linked to biological effects [13]. Although ATRA and 9/13cis RA were already reported in pioneer studies focused on retinoids in freshwater blooms [8,10], the first occurrence of 5,6epoxy-ATRA in aquatic ecosystems was reported only recently [11].

Furthermore, both experiments also showed ATRA hydroxylation in a variant containing MES buffer with H_2_O_2_ which led to the production of 4OH-ATRA. It pointed to the involvement of H_2_O_2_ in the production of this bioactive ATRA metabolite. Besides, the ATRA hydroxylating enzyme CYP120A1 has been already described in cyanobacteria [25], but it catalyzes hydroxylation at positions 2, 16, and 17 in the ATRA molecule, not in position 4. Nevertheless, the presence of these enzymatically synthesized molecules in cyanobacterial biomass has not been studied so far. In addition, our results documented an increase in 4keto-RAL in all variants. On the other hand, only the Fenton reaction showed the production of 13cis RA methyl ester that was measured in earlier studies but not detected in the environmental samples, nor in the cyanobacterial cultures.

The strongest oxidation force is evolved by the Fenton reaction, where the hydroxyl radical (∙OH), superoxide radical (O_2_^−^), and singlet oxygen (^1^O_2_) are produced by the reaction of H_2_O_2_ and iron [26]. It correlates well to the level of produced retinoids, since the most significant RAL oxidation was observed during the Fenton reaction that resulted in the highest total concentration of the analyzed retinoid molecules (Figure 4 and Supplementary Materials S4 and S5 ). The increase in the retinoid concentration in individual variants reflected the amount of ROS available for RAL oxidation, as described in the Methods section—Experimental design (2.7.1). Moreover, what is important in terms of environmental relevance is that our experiments showed the production of more bioactive retinoids from RAL by ROS in water. This can pose a risk, especially in waterbodies with a massive development of water blooms. Furthermore, our results correspond to the previous reports that MES decreases the level of available ROS [27,28]. It can be observed from the comparison between the water variant and the MES variant. Since the availability of ROS is important also for the Fenton reaction, the ratio of reagents for the Fenton reaction has been optimized (Materials and Methods—2.7.2 and Supplementary Material S6).

In addition to the level and spectra of the detected retinoid compounds, the reported bioactivity provided valuable information allowing an for extrapolation of biological effects (Figure 4). The results showed the highest increase in bioactivity in the Fenton reaction, followed by the water variant, and statistical analysis confirmed the significant increase in activity in these two variants in comparison to all (control) MES variants. Although only selected retinoids were analyzed within this study, the reaction of RAL with ROS can result in much broader spectra of molecules, which can influence the final detected bioactivity. Importantly, it was described that structurally very similar compounds to retinoids, 14´-β-apocarotenoid and 13-β-apocarotenoid, compete with ATRA for the ligand-binding domain in the RAR receptor [6]. Our results indicate similar properties for RAL oxidation products; therefore, wider spectra of free radical oxidation products of RAL would have to be analyzed to fully explain the observed bioactivities. Moreover, our earlier study focused on freshwater blooms dominated by cyanobacteria showed that the contents of currently studied retinoid representatives did not explain the retinoid-like activity at most localities [11].

### 2.4. Production of Retinoids and Biological Function

The production of retinoids in cyanobacteria starts from the biosynthesis of RAL. Both enzymatic and non-enzymatic production of RAL from carotenoids or apocarotenoids was shown [15,16,19]. Importantly, RAL can be enzymatically produced from various apocarotenoids substrates containing C15, the C15´ double bond by ACO [15,29], which emphasizes RAL’s importance in cyanobacterial cells, where it is used in energy production in complex with rhodopsin. Regarding further metabolism of RAL into retinoid products observed in cyanobacteria, our study pointed out that the production of biologically active retinoids can be mediated via ROS, although possible enzymatic biosynthesis of some other retinoids has also been shown in cyanobacteria *Synechocystis* [25].

**Figure 5:**
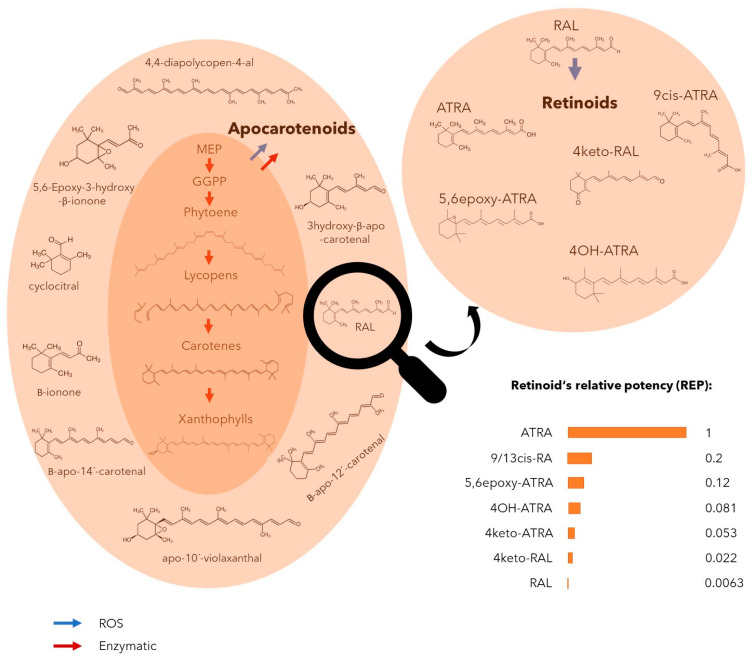
Illustrated origin of retinoid production from carotenoids controlled by enzymatic and non-enzymatic reactions. Relative potency (REP) of individual retinoids to interact with a retinoic acid receptor (RAR) is expressed as values from 0–1 where REP of all-trans retinoic acid (ATRA) means maximal value [20]. Blue arrow indicates steps catalyzed non-enzymatically by reactive oxygen species (ROS). Red arrow indicates steps catalyzed enzymatically. Abbreviations: Retinal (RAL), methylerythritol phosphate (MEP) pathway, geranylgeranyl pyrophosphate (GGPP).

In the case of the most bioactive retinoid ATRA, we showed that the enzyme possibly responsible for ATRA production is absent across many cyanobacterial genomes. Simultaneously, our results showed that ATRA is the first and the main product of RAL freeradical oxidation observed in our experiments. Another directly detected product of RAL oxidation was 4keto-RAL; nevertheless, other observed molecules are known to be produced by the consequent oxidation (5,6epoxy-ATRA) and hydroxylation (4OH-ATRA) of ATRA. Special molecules are 9cis-RA and 13cis-RA, which have identical fragmentation patterns on LC-MS-MS and can be produced by isomerization from both RAL and ATRA. Based on these results, we identified the origin and production of retinoids in cyanobacteria (Figure 5), although it can be anticipated that these reactions are widespread across other organisms producing retinal such as bacteria, algae, or fungi, and much broader spectra of retinoid-like molecules can be produced by ROS mediated reactions of carotenoids.

Regarding retinoids’ biological function, it is well known that RAL serves as a chromophore in complex with rhodopsin, which is an important part of energy metabolisms in bacteria, cyanobacteria, or fungi. However, our results also indicate a function of retinoids as antioxidants or stress signals in cyanobacteria. Generally, carotenoids are well-known antioxidants due to their conjugated double bonds [19]. Importantly, it has been shown that β-carotene and its oxidation products trans-β-8´-apocarotenal and β-ionone elicit antioxidant properties, although the chain length of these molecules significantly differs [30]. It suggests that the antioxidative effects of these molecules are dependent more on the presence of conjugated double bonds than chain length. Therefore, since RAL, as well as ATRA, both contain conjugated double bonds, the function of these molecules in cyanobacteria as ROS quenchers can be anticipated. Moreover, given that RAL production is widespread across the tree of life, retinoids´ function as an auxiliary ROS quencher can be very conserved across various groups of organisms such as bacteria, fungi, or plants in which a significant amount of ROS is emitted during membrane processes. In addition, it has been reported that free radical-mediated oxidation products can serve as important stress signals, which has been already described for carotenoid oxidation products such as β-ionone or β-cyclocitral [31].

## 3. Conclusions

Our study showed that the production of retinoid-like compounds is undoubtedly associated with cyanobacteria, as their production and bioactivity were confirmed in the axenic cyanobacterial cultures. A key step in the production of retinoids is the oxidation of carotenoid product RAL to ATRA and our bioinformatic analysis did not reveal any possible enzyme responsible for this reaction across cyanobacterial genomes. Our consequent experiments focused on non-enzymatic, free radical-mediated oxidation of RAL, and identified that it leads to the production of more bioactive products that can interact with retinoid receptors. The production of these metabolites by cyanobacteria in association with the mass development of water blooms can have adverse biological consequences in aquatic ecosystems regarding the described teratogenic effects of retinoids, especially in areas where the occurrence of water blooms coincides with the early development of aquatic vertebrates and invertebrates. Moreover, our findings that RAL can be converted by ROS into more bioactive retinoids also in water, and out of the cells, increases the environmental significance of this process. To conclude, the identification of the non-enzymatic production of these bioactive metabolites provides important information, which extends our knowledge about the metabolic processes in cyanobacteria and can be further used in risk assessment.

## 4. Materials and Methods

### 4.1. Cultivation of Axenic Cyanobacterial Cultures

The used axenic cyanobacterial strains originated from two different culture collections: (1) The Pasteur Culture Collection of Cyanobacteria, Paris, France (*Aphanizomenon flos-aquae* PCC 7905, *Planktothrix agardhii* PCC 7805, *Microcystis aeruginosa* PCC 7806,), and (2) The Culture Collection of Algae at the University of Texas, Austin, USA (*Anabaena flos-aquae* UTEX 1444). All species were cultivated in a 1:1 (*v*/*v*) mixture of Zehnder medium [32] and Bristol Bold medium [33] at 22 °C under continuous light, with a photosynthetic photon flux density (PPFD) of 26.99 umol/s/m2. After 21 days of cultivation, cells were harvested by centrifugation (5 min, 2630× *g*). The axenicity of the cultures was routinely checked using R2A agar cultivation as previously reported [34] and also by 16S amplicon sequencing (as described below). Harvested cells were freeze-dried and extracted for chemical analyses as described below.

### 4.2. 16S Amplicon Sequencing

#### 4.2.1. DNA Extraction

The extraction of DNA from the biomass of the analyzed cyanobacterial strains was conducted based on Morin et al. [35]. Briefly, 20 mg of freeze-dried biomass was resuspended in 0.5 mL of 0.15 M NaCl/0.1M EDTA solution and homogenized using three freeze-thaw cycles with liquid nitrogen. Then, samples were centrifuged (10 min, at 7200× *g*), the supernatant discarded, the pellet resuspended in 0.5 mL of TE buffer, and then 1 µL of RNase was added and the samples were incubated at 37 °C. After 1 h incubation, 100 µL of lysozyme (50 mg/mL) was added and samples were incubated at 37 °C. After 30 min incubation, 5 µL (50 mg/mL) of protein kinase K and 2% (final concentration) of sodium dodecyl sulfate (SDS) was added, and samples were incubated at 55 °C for 1 h. Then, selective precipitation was performed by adding 150 µL 5M NaCl to the tubes followed by 0.1 volume (of total volume) of 10% CTAB (cetyltrimethylammonium bromide) stock solution, mixed by inversion, and incubated at 65 °C for 10 min. For purification, 1 volume of chloroform was added, and tubes were incubated on ice for 30 min to allow for protein precipitation. Then, samples were centrifuged (10 min, 7200× *g*, 4 °C), the supernatant transferred to fresh tubes, mixed with 0.6 volume of isopropanol, and incubated overnight at 4 °C. Following this, samples were centrifuged (30 min, 16,000× *g*, 4 °C), isopropanol supernatant was discarded, and the pellet was washed with 1 mL of 70% ethanol. Finally, samples were centrifuged (30 min, 16,000× *g*, 4 °C), the supernatant discarded, the pellet air-dried, and then resuspended in 50 µL of TE buffer. The concentration and quality of the extracted DNA were checked via NanoDrop (ThermoFisher Scientific, Waltham, MA, USA, N.A.) and agarose gel electrophoresis.

#### 4.2.2. Illumina Library Preparation

The extracted DNA was used as a template in PCR targeting the hypervariable region of the bacterial 16S rRNA gene. The metagenomic library was prepared according to the Illumina 16S Metagenomic Sequencing Library Preparation protocol, with some deviations as described in Appendix A. Sequencing was performed with the MiSeq reagent kit V2 using MiSeq instrument in accordance with the manufacturer’s instructions (Illumina, USA).

#### 4.2.3. Bioinformatic Processing of 16S rRNA Data

Paired reads from 16S rRNA sequencing were first processed using an in-house pipeline implemented in Python 3. The steps of processing included the trimming of low-quality 3′ ends of reads, removal of read pairs containing unspecified base N, and removal of pairs containing very short reads. A detailed description of library preparation, sequencing, and data processing is in Appendix A.

### 4.3. Search for Enzyme Converting RAL to ATRA

Since the enzyme responsible for the conversion of RAL to ATRA was reliably identified only in some vertebrates including humans, we used BLAST [36] to search for similar proteins across cyanobacterial genomes. We decided to search for all aldehyde dehydrogenases (aldh) across cyanobacterial genomes which belong to the same protein SF as retinal dehydrogenase from *Homo sapiens* in order to attain a deeper insight into the distribution and evolution of individual aldh across cyanobacterial lineage.

### 4.4. Distribution of Aldh across Cyanobacterial Genomes

Based on the NCBI Taxonomy database of cyanobacteria, at least one representative genus from each class with an available genome, together with representative species, was selected. Across these genomes, a BLAST search for all aldh belonging to SF-family aldh was carried out. Only aldh with a specific conserved domain (CD) distributed across at least five cyanobacterial genomes were considered for analysis. Phylogenetic analysis of cyanobacteria based on 16S rRNA gene was carried out according to Shirmeister et al. [37]

### 4.5. Phylogenetic Analysis of Aldh

To study the functional similarity and evolution of cyanobacterial aldh belonging to the same SF as retinal dehydrogenase from *Homo sapiens*, a phylogenetic analysis was carried out. Across the cyanobacterial genomes available at the NCBI (the end of March 2021), aldh belonging to 10 different conserved domains was found. For each conserved domain, five representative sequences from different cyanobacterial species were used for alignment and phylogenetic tree building. The alignment was created using ClustalW aligner within MEGA X [38]. The evolutionary history was inferred by using the maximum likelihood method and JTT matrix-based model [39]. The bootstrap consensus tree inferred from 1000 replicates was taken to represent the evolutionary history of the taxa analyzed [40]. Branches corresponding to partitions reproduced in less than 50% bootstrap replicates were collapsed. Initial tree(s) for the heuristic search were obtained automatically by applying the Neighbor-Join and BioNJ algorithms to a matrix of pairwise distances estimated using the JTT model, and then selecting the topology with a superior log-likelihood value. A discrete Gamma distribution was used to model evolutionary rate differences among sites (five categories (+G, parameter = 1.7152)). The rate variation model allowed for some sites to be evolutionarily invariable ([+I], 0.33% sites). This analysis involved 55 amino acid sequences. There were in total 460 positions in the final dataset. The final phylogenetic tree was visualized using iTOL [41].

### 4.6. Biomass Extraction

Biomass extracts were prepared according to the extraction procedure described earlier in Javůrek et al. [8] with slight modifications: 100 mg of freeze-dried biomass was disintegrated by sonication in a glass test tube with 5 mL of methanol in a cooling bath. After sonication, extracts were centrifuged, and the liquid phase was transferred to glass vials. The final concentration was adjusted to 400 g dry mass (dm)/L under nitrogen.

### 4.7. Fenton Reaction

#### 4.7.1. Experimental Design

All reaction variants contained two basic components, distilled water and retinal. Distilled water naturally contains a low level of ROS [42]. Moreover, Blough and Zepp [43] reported that the absorption of light by organic chromophores (e.g., retinal, in our case) within water initiates a cascade of reactions producing ROS. Although the reaction mixtures were prepared in dark vials, it is impossible to completely prevent light absorption during the experiment. Therefore, all variants contained a background level of ROS. To characterize the impact of various levels of ROS on retinal degradation, variants with increased and decreased levels of ROS were designed. To produce more ROS, the Fenton reaction was used which has been described earlier (e.g., [44]). To decrease the level of ROS, MES buffer was used since it was reported to inhibit the production of ROS similarly to phosphate, HEPES, or MOPS buffers [27,28]. Simultaneously, MES buffer is a necessary component of the Fenton reaction to retain an optimal pH for ROS production. In total, five experimental variants were designed: (1) Fenton reaction—increased ROS level, (2) Water—unaffected, background level of ROS, (3) MES—decreased level of ROS, (4) MES with Fe+—decreased level of ROS affected by the presence of ferrous iron, and (5) MES with H_2_O_2_—MES with a decreased level of ROS affected by the addition of hydrogen peroxide. Such an experimental design allowed for the evaluation of the influence of the ROS level on retinal degradation and the production of more bioactive retinoids, as well as the effect of the Fenton reaction and its individual components.

#### 4.7.2. Experimental Set-Up

To set up the optimal conditions for the investigation of dynamics of RAL oxidation, a ratio of Fenton´s reagents FeSO4 and H_2_O_2_ was optimized. Four molar ratios were tested in two independent experiments, and the optimal ratio for product detection was selected for the main experiment (Supplementary Material S6).

In detail, 2 mM stock solutions of FeSO4 (Fe stock) and H_2_O_2_ (H_2_O_2_ stock) diluted in 20 mM MES buffer (pH = 5) were prepared. Consequently, 495 µL Fe stock was mixed with 10 µL 1 mM RAL in 100% MeOH. Finally, the Fenton reaction (1) was started by the addition of 495 µL H_2_O_2_ stock. The final mixture was incubated at room temperature for 24 h. Other experimental variants consisted of 10 µM RAL in (2) distilled water, (3) MES buffer (pH = 5), (4) in MES buffer with 1 mM FeSO4 (pH = 5), (5) in MES buffer with 1 mM H_2_O_2_ (pH = 5), respectively. The 250 µL fractions from all variants were collected at times 0, 2, 4, and 24 h. The oxidation in each collected fraction was stopped by the addition of one NaOH pellet, heating to 90 °C for 5 min, and cooling down, and the reaction was diluted with the addition of 250 µL 100% MeOH. Two independent experiments were carried out and each tested variant was performed in triplicates.

### 4.8. Retinoid-like Activity Analysis

The retinoid-like activity was analyzed using the RARα Reporter (Luc)-HEK293 Cell Line (Catalog number: 60503, BPS Bioscience Inc., San Diego, CA, USA) containing a firefly luciferase gene under the control of retinoic acid response elements stably integrated into HEK293 cells along with full-length human RARα (NM_000964). The method is described in detail in an earlier study [11]. Detailed information about the used cell line is available on the websites of the manufacturer (BPS Bioscience Inc., USA).

### 4.9. LC-MS/MS Analysis of Retinoids

A detailed description of analytical methods for the detection of retinoids is given in Appendix A. The sensitive LC-MS/MS method was used for the analysis of ATRA, RAL, 9/13cis-RA, 4keto-9cis-RA, 4keto-13cis-RA, 4keto-ATRA, 4keto-RAL, 5,6epoxy-ATRA, 4OH-ATRA, and 13cis-methylester-RA. MS parameters for the analyzed retinoid compounds are shown in the Supplementary Information (Table S3).

### 4.10. Data Analysis

Statistical analysis of data was carried out using GraphPad Prism. For a comparison of the statistical differences between retinoid-like activities of axenic and non-axenic cultures, the *t*-test method was used. Statistical significance of the differences among treatments in oxidation experiment by ROS was analyzed using one-way ANOVA.

The average values of concentration of retinoid compounds across the replicates of the same treatment variant within one experiment were calculated in case at least one of the triplicates was above LOQ. For this purpose, values under LOQ (NQ) were substituted by LOQ/2. The values under LOD were replaced by fixed value LOD/2 for data analysis.

Retinoid equivalents (REQ) were calculated using the program GraphPad Prism. Relative luminescence units obtained from in vitro cellular reporter assay were converted to the percent of the maximum response of the ATRA standard curves (ATRAmax; 100%). For the comparison of retinoid-like activities of axenic and non-axenic cultures, REQ were calculated by relating the ECX value of standard calibration to the concentration of the tested sample inducing the same response [45]. EC20 values were calculated from a nonlinear logarithmic regression of the dose–response curves of ATRA calibration and samples using GraphPad Prism (GraphPad Software, San Diego, CA, USA). For samples whose response did not reach 20% ATRAmax, the EC10 values were used for the calculations.

## Data Availability

All data are available within main text or in Supplementary Materials.

## Supplementary Materials

The following are available online at https://www.mdpi.com/article/10.3390/toxins14090636/s1, Supplementary material S1 contains Excel sheet S1a: Retinoids concentration in cultures of axenic cyanobacteria, Excel sheet S1b: Taxonomic composition of cultures of axenic cyanobacteria based on 16S data. Supplementary material S2 contains Excel sheet S2: Cyanobacterial ALDHs—sequences. Supplementary material S3 contains Excel sheet S3: Cyanobacterial 16S rRNA—sequences. Supplementary material S4 contains Excel sheet S4: Exp 1 Data—Concentration, REQchem. Supplementary material S5 contains Excel sheet S5: Exp 2 Data—Concentration, REQchem. Supplementary material S6 contains Excel sheet S6: Optimization of Fenton reaction. Supplementary material—Tables S1–S4 contain Table S1: ALDH—BLAST comparison, Table S2: Basic characteristics of analyzed cyanobacterial aldehyde dehydrogenases, Table S3: MS parameters for analyzed retinoid compounds, and Table S4: Primers and PCR.

## Appendix A

Illumina library preparation

Extracted DNA was used as a template in PCR targeting the hypervariable region of the bacterial 16S rRNA gene. A metagenomic library was prepared according to the Illu-mina 16S Metagenomic Sequencing Library Preparation protocol with some deviations. Each PCR was performed with the Phusion Hot Start II High-Fidelity PCR Master Mix (ThermoFisher Scientific, Waltham, MA, USA) in triplicate, with the primer pair consist-ing of Illumina overhang nucleotide sequences, an inner tag, and gene-specific sequences [46,47,48]. The Illumina overhang served to ligate the Illumina index and adapter. Each in-ner tag (a unique sequence of 7–9 bp) was designed to differentiate samples into groups. Primer sequences and PCR cycling conditions are summarized in Table S4. After PCR amplification, triplicates were pooled, and the amplified PCR products were visualized by gel electrophoresis. PCR product purification was performed with Agencourt AMPure XP beads (Beckman Coulter Genomics). Samples with different inner tags were equimolarly pooled based on fluorometrically measured concentration using Qubit dsDNA HS Assay Kit (Invitrogen, ThermoFisher Scientific, Waltham, MA, USA) and microplate reader (Bi-oTek Synergy, Agilent Technologies, Santa Clara, CA, USA). Pooled PCR products were used as a template for a second PCR with Nextera XT indices (Illumina, San Diego, CA, USA). Differently indexed samples were quantified using the qPCR KAPA Library Quanti-fication Complete Kit (Roche, Basel, Switzerland) and LightCycler 480 Instrument (Roche, Basel, Switzerland) and equimolarly pooled according to the measured concentration. The prepared libraries were checked with a 2100 Bioanalyzer Instrument using the High Sen-sitivity D5000 Screen tape (Agilent Technologies, Santa Clara, CA, USA) and the DNA concentration was measured with qPCR shortly prior to sequencing. The final library was diluted to a concentration of 8 pM and 20% of PhiX DNA (Illumina, San Diego, CA, USA) was added.

Bioinformatic processing of 16S rRNA data

Paired reads from 16s rRNA sequencing were first processed using an in-house pipe-line implemented in Python 3. The steps of processing included the trimming of low-quality 3′ ends of reads, the removal of read pairs containing unspecified base N, and the removal of pairs containing very short reads. In order to minimize the sequencing and PCR-derived errors, forward and reverse reads were denoised using the DADA2 amplicon denoising R package [49]. Following denoising, the forward and reverse reads were joined into a single longer read using the fastq-join read joining utility [50]. To be joined, reads in pairs had to have an overlap of at least 20 base pairs with no mismatches allowed. Pairs in which this was not the case were discarded. As the final step, chimeric sequences were removed from the joined reads using the removeBimera function of the DADA2 R pack-age. Subsequent taxonomic assignment was conducted by the uclust-consensus method from the QIIME [47] microbial analysis framework using the Silva v. 123 [51] reference database.

List of chemicals and reagents

Standard all-trans-retinoic acid (ATRA, ≥98%), 13-cis-retinoic acid (13-cis RA, ≥98%) were obtained from Sigma-Aldrich (Merck, Kenilworth, NJ, USA), 9-cis-retinoic acid (9-cis RA, ≥98%) from Santa Cruz Biotechnology, and all trans-retinal (RAL), rac all- trans 4-hydroxy retinoic acid (4OH-ATRA), all-trans 4-keto retinoic acid (4keto-ATRA), 4-keto retinal (4keto-RAL), all-trans 5,6-epoxy retinoic acid (5,6epoxy-ATRA), 4-keto 13-cis-retinoic acid (4keto-13cis-RA), and 4-keto 9-cis-retinoic acid (4keto-9cis-RA) from Toronto Research Chemicals (Ontario, ON, Canada)., which do not provide % purity. La-beled standards of ATRA-d5, retinal-d5, 4keto-ATRA -d3, and 4keto-13cis-RA -d3 were purchased from Toronto Research Chemicals (Ontario, ON, Canada).

LC-MS/MS analysis of retinoids

Analysis was performed with the Waters LC chromatograph (Waters, Manchester, UK) and the chromatographic separation was achieved using a column Acquity BEH C18 of 100 × 2.1 mm ID and a 1.7 μm particle size. A gradient elution method was used with phase A (0.1% formic acid in water) and phase B (0.1% formic acid in acetonitrile). MS grade acetonitrile and formic acid (FA, 99%) were purchased from BIOSOLVE BV (Valkenswaard, Netherlands). The gradient elution started with 20% B, increased to 70% B over 1 min, and to 100% B from 1 to 5 min, and was held at 100% B from 5 to 7 min, then decreased to 20% B in 7 min, and then again was held for 2 min to equilibrate the system before the next injection. The flow rate was set at 0.3 mL/min and the injection volume at 7 μL. The column and sample temperatures were set at 40 °C and 10 °C, respectively. Detection was performed on a Xevo TQ-S quadrupole mass spectrometer (Waters Manchester, UK) and analytes in the ESI+ mode were detected using tandem mass spec-trometry. The collision energy of transitions (ATRA, 9cis-RA, 13cis-RA, retinal, 4keto-atRA, 13-cis-oxoRA, 9-cis-oxoRA, 5,6epoxy-atRA, and 4keto-retinal; 9-cis RA and 13-cis RA were reported as a sum) were optimized for each analyte (Table S1). The capil-lary voltage was set at 0.8 kV and the cone, desolvation, and collision gas flows were set at 150 L/h, 600 L/h, and 0.15 mL/min, respectively. The source and desolvation temperatures were set at 150 °C and 500 °C, respectively. Data were processed by MassLynxTM software (Manchester, UK). The quantification of analytes was based on the most intensive MRM transition, considering relative signals between each analyte and internal standard (4keto atRA-D3; retinal-D5; atRA-D5; 13cis-oxoRA-D3). The limit of detection (LOD, signal to noise ratio S/N>3) and quantification (LOQ, S/N>10) were 0.5 and 1 ng/mL, respectively, for each compound.
