# Supplementary material for: The Origin of Teratogenic Retinoids in Cyanobacteria
Source: Toxins (Basel). 2022 Sep 15;14(9):636. doi: 10.3390/toxins14090636 (PMC9501733; doi:10.3390/toxins14090636)
Supplement: Supplementary file 1 [file toxins-14-00636-s001.zip › toxins-1888720-supplementary.pdf]

SUPPLEMENTARY INFORMATION TO PAPER:

The origin of teratogenic retinoids in cyanobacteria

Authors: Luděk Sehnal, Marie Smutná, Lucie Bláhová, Pavel Babica, Petra Šplíchalová, Klára Hilscherová

Table S1. ALDH - BLAST comparison. Similarity comparison of cyanobacterial and human enzymes containing CD F1-2. Part A shows basic information about enzyme annotation and origin. Part B shows BLAST comparison of individual enzymes. Abbreviations: C – percentage (%) cover of compared sequences; E – E-value; S - percentage (%) similarity.

**A**	**Accession number**	**Species**	**Enzyme**	**Conserved Domain**
**ToB**	WP_038075203.1	*Tolypothrix_bouteillei*	ALDH	F1-2
**CroCh**	WP_008272605.1	*Crocosphaera_chwakensis*	ALDH	F1-2
**H-A1**	NP_000680.2	*Homo_sapiens*	ALDH A1	F1-2
**H-A2**	NP_003879.2	*Homo_sapiens*	ALDH A2	F1-2
**H-2**	NP_000681.2	*Homo_sapiens*	ALDH 2	F1-2

**B**	**ToB**	**CroCh**	**H-A1**	**H-A2**	**H-2**
**ToB**		C100%; E0.0; S82.7%	C98%; E0.0; S63.7%	C97%; E0.0; S62.8%	C98%; E0.0; S67.4%
**CroCh**	C100%; E0.0; S82.7%		C98%; E0.0; S64.8%	C98%; E0.0; S63.7%	C98%; E0.0; S68.9%
**H-A1**	C98%; E0.0; S63.7%	C98%; E0.0; S64.8%		C98%; E0.0; S73.2%	C100%; E0.0; S68.1%
**H-A2**	C97%; E0.0; S62.8%	C98%; E0.0; S63.7%	C98%; E0.0; S73.2%		C96%; E0.0; S67.3%
**H-2**	C98%; E0.0; S67.4%	C98%; E0.0; S68.9%	C100%; E0.0; S68.1%	C96%; E0.0; S67.3%	

Table S2. Basic characteristics of analyzed cyanobacterial aldehyde dehydrogenases. Only one example sequence of aldehyde dehydrogenase for each conserved domain is presented.

Accession number	Organism	Enzyme	Conserved domain	Product	Function
WP_042155384.1	Planktothrix agardhii	CoA-acylating methylmalonate-semialdehyde dehydrogenase	ALDH_F6_MMSDH	propionyl-CoA	Amino acid metabolism - catabolism
WP_067774485.1	Nostoc sp. NIES 3756	proline/L-glutamate gamma-semialdehyde dehydrogenase	Pro_dh + ALDH_PutA	glutamate	two step catabolism of proline to glutamate
WP_067771050.1	unclassified Nostoc	aldehyde dehydrogenase family protein	CAJ73105	uncharacterized	-
WP_067769491.1	Nostoc sp. NIES 3756	glutamate-5-semialdehyde dehydrogenase	ProA	L-glutamate 5-semialdehyde and phosphate	L-proline biosynthesis
WP_067769931.1	Nostoc sp. NIES 3757	aldehyde dehydrogenase family protein	F1-2	retinoic acid; tetrahydrofolate and CO2; acetate	oxidation of retinal; 10-formyltetrahydrofolate; acetaldehyde
ARI84357.1	Microcystis aeruginosa PCC 7806SL	NAD-dependent succinate-semialdehyde dehydrogenase	ALDH_SSADH1_GabD1	succinate	oxidation of succinate semialdehyde to succinate in the degradation of glutamate and 4-aminobutyrate
OBQ44124.1	Aphanizomenon flos-aque WA102	aldehyde dehydrogenase	YwdH	uncharacterized	oxidation of both apocarotenals and alkanals into corresponding acids
		aldehyde dehydrogenase	CALDH	apocarotenoid acid >C25	oxidation of both apocarotenals and alkanals into corresponding acids
WP_096594581.1	Calothrix sp. NIES 2098	aldehyde dehydrogenase family protein	DDAldH	4,4'-diapolycopene-diacid	4,4'-diapolycopene-dialdehyde into 4,4'-diapolycopene-diacid

Table S3. MS parameters for analyzed retinoid compounds; MRM (multiple reaction monitoring); quantification ions marked (bold).

Compound	MRM transition	Cone voltage (V)	Collision energy (V)
4OH-ATRA	**299.2 → 95.2**	30	23
	299.2 → 157.2	30	25
4keto-ATRA	**315.2 → 137.1**	30	25
	315.2 → 241.2	30	15
4keto-RAL	**299.2 → 147.2**	30	24
	299.2 → 189.2	30	16
5,6epoxy-ATRA	**317.2 → 107.2**	30	21
	317.2 → 153.2	30	14
9/13*cis*-RA	**301.2 → 159.1**	30	23
	301.2 → 205.1	30	13
ATRA	**301.2 → 159.1**	30	23
	301.2 → 205.1	30	13
RAL	**285.3 → 161.2**	30	9
	285.3 → 175.2	30	13
4keto-13*cis*-RA	**315.3 → 137.1**	30	25
	315.3 → 241.2	30	15
4keto-9*cis*-RA	**315.3 → 137.1**	30	25
	315.3 → 241.2	30	15
ATRA-d5	**306.2 → 162.2**	30	20
	306.2 → 206.2	30	15
RAL-d5	**290.3 → 161.2**	30	8
	290.3 → 180.3	30	15
	290.3 → 198.2	30	7
4keto-ATRA-d3	**318.2 → 137.2**	30	24
	318.2 → 162.2	30	14
	318.2 →244.2	30	14
4keto-13*cis*-RA-d3	**321.2 →143.1**	30	25
	321.2 →247.2	30	15

Table S4. **Primers and PCR**. Sequences of used primers and PCR conditions for Illumina library preparation.

	Primer		Sequence	Product size	Reference
**V4 16S rRNA gene**	**16S_F**	forward	TCGTCGGCAGCGTCAGATGTGTATAAGAGACAG-InnerTag-GTGYCAGCMGCCGCGGTAA	~ 300	*Caporaso et al., 2011; Apprill et al., 2015*
	**16S_R**	reverse	GTCTCGTGGGCTCGGAGATGTGTATAAGAGACAGC-InnerTag-GGACTACNVGGGTWTCTAAT		

**V4 16S rRNA gene**<br>Thermal cycling conditions		
Temperature	Time	cycle
95°C	30 s	
98°C	10 s	
55°C	15 s	30x
72°C	30 s	
72°C	10 min	
10°C	10 min	

References:

Apprill, A., Mcnally, S., Parsons, R., & Weber, L. (2015). Minor revision to V4 region SSU rRNA 806R gene primer greatly increases detection of SAR11 bacterioplankton. *Aquatic Microbial Ecology*, *75*(2), 129–137. https://doi.org/10.3354/ame01753

Caporaso, J. G., Lauber, C. L., Walters, W. A., Berg-Lyons, D., Lozupone, C. A., Turnbaugh, P. J., Fierer, N., & Knight, R. (2011). Global patterns of 16S rRNA diversity at a depth of millions of sequences per sample. *Proceedings of the National Academy of Sciences of the United States of America*, *108*(SUPPL. 1), 4516–4522. https://doi.org/10.1073/pnas.1000080107

List of other Supplementary materials (attached MS Excel files)

Supplementary material S1

Excel sheet S1a: Retinoids concentration in cultures of axenic cyanobacteria

Excel sheet S1b: Taxonomic composition of cultures of axenic cyanobacteria based on 16S data

Supplementary material S2

Excel sheet: Cyanobacterial ALDHs - sequences

Supplementary material S3

Excel sheet: Cyanobacterial 16S rRNA - sequences

Supplementary material S4

Excel sheet: Exp 1 Data – Concentration, REQchem

Supplementary material S5

Excel sheet: Exp 2 Data – Concentration, REQchem

Supplementary material S6

Excel sheet: Optimization of Fenton reaction
